# Research on grandchild care and depression of chinese older adults based on CHARLS2018: the mediating role of intergenerational support from children

**DOI:** 10.1186/s12889-022-12553-x

**Published:** 2022-01-19

**Authors:** Shaoliang Tang, Tongling Yang, Chaoyu Ye, Meixian Liu, Ying Gong, Ling Yao, Yun Xu, Yamei Bai

**Affiliations:** 1 School of Health Economics and Management, Nanjing University of Chinese Medicine, Nanjing, China; 2 School of Nursing, Nanjing University of Chinese Medicine, Nanjing, China

**Keywords:** Grandchild care, depression, intergenerational support, mediating effect

## Abstract

**Background:**

There may be differences in gender and marital status in the impact of grandchild care on the depression of the Chinese older adults. This research explores the effect of grandchild care on the depression of Chinese older adults of different genders and marital status, and explores the mediating role of intergenerational support from children between grandchild care and depression.

**Methods:**

This research uses the data of 3540 Chinese older adults from the China Health and Retirement Longitudinal Study (CHARLS) in 2018. The OLS model is used to analyze the effect of grandchild care on the depression of the older adults. and the older adults are classified according to gender and marital status, and the differences in the effect of grandchild care on the depression of the elderly of different genders and marital status is explored. Finally, the bootstrap method is used to test the mediating effect of intergenerational support from children.

**Results:**

The research finds that grandchild care has a significant impact on the depression of the older adults in China, and providing grandchild care can significantly reduce the depression of the older adults. The effect of grandchild care on the depression of the older adults is different between different genders and marital status. After categorizing the older adults by gender, the grandchild care only has a significant impact on the depression of female older adults; after classified by marital status, grandchild care only has a significant impact on the depression of the older adults who don’t have a spouse. The mediating effect analysis shows that both children’s emotional support and children’s economic support have a mediating effect between grandchild care and depression of the older adults.

**Conclusion:**

The depression of the Chinese older adults is affected by grandchild care, and this effect is more prominent in female older adults and the older adults who don’t have a spouse. The society should support and encourage capable older adults to participate in grandchild care, and children should also provide more intergenerational support to the older adults who provide grandchild care, so as to further play the role of grandchild care in relieving depression of the older adults.

## Background

Since the 21st century, the proportion of the older adults population in China has been on the rise, and the degree of aging is getting higher [1–3]. According to China’s latest national census, people over 60 accounted for 18.7% of China’s total population, and this is an increase of 5.44% compared to the previous census [4]. For many countries, the older adults are the main consumers of medical resources, and the health problems of older adults have become a hot spot of the whole society and the main targets of various countries’ health policies[3] [5, 6]. The older adults are the high-risk group of mental health, and depression is the most common mental illness in the older adults, which will increase the risk of suicide and death of them [7–9]. In today's call for healthy aging, the problem of depression of the older adults has become a major research area, and alleviating the depression of the older adults has become one of the ways to achieve healthy aging [10].

Grandchild care is an important factor that affects depression of the older adults, and its situation differs in different cultural backgrounds and countries [11, 12]. Hank, K found from a survey on Europe that the ten European countries are highly involved in grandchild care, but there are significant differences in the prevalence and intensity of their grandchild care due to different policies for child care and (mother or female) employment systems [13]. Laughlin’s research pointed out that in the United States, one out of four children under the age of five is taken care of by grandparents [14]. However, grandparents provide care mostly because their children are in special periods such as divorce, poverty, and drug abuse, or grandchildren are abused and neglected by their children, namely the older adults' children [15]. In East Asian countries with heavy family concepts, it is more common for the older adults to provide grandchild care. They do not regard caring for their grandchildren as a burden, but as a way to realize their self-worth [16]. In China, social child care services are not yet complete. Taking care of children has become the task of grandparents. Especially since the implementation of the relaxation of the fertility policy, whether grandparents provide grandchild care services will greatly affect the childbirth decision of adult children [17].

## Grandchild care and depression of older adults

Academia has not yet reached a consensus on the impact of grandchild care on depression of older adults, but the relationship between grandchild care and the health of the older adults is mostly explored from the Role Strain Theory and Role Expansion Theory. The role strain theory believes that individuals will experience the negative effects of multiple roles, especially when the individual’s multiple roles conflict with each other, which will cause pressure [18]. From this research perspective, providing grandchild care will squeeze the time of their own health investment, will increase the physical burden, and will lose the older adult's own activity arrangements, which makes the older adults who provide grandchild care have a depressive tendency [19, 20]. On the contrary, the role expansion theory believes that individuals can obtain social integration and satisfaction from multiple roles, adding color and vitality to life [21]. According to this theory, providing care will increase the chances of grandparents’ social participation, makes the grandparents obtain more social support, especially from their adult children, improves the relationship with children, and enhances the subjective health and life satisfaction of grandparents [22, 23]. These are beneficial to the mental health of older adults and achieve the effect of improving depression.

Taking care of grandchildren is a social participation with Chinese cultural character, one of the important family characters of the older adults in China [24]. The actual impact of care behavior on the mental health of older adults is related to the characteristics of the older adults, the characteristics of the grandchildren, and the characteristics of the family, and it is necessary to conduct further empirical tests with national large-scale public data.

### Grandchild care and depression in different older adults groups

Previous scholars had explored the influence of grandchild care on the physical and mental health of the older adults, but did not consider the difference between different older adults groups [25, 26]. According to the previous studies about grandchild care, we can find that female older adults were more frequently involved in the grandchild care, and male older adults usually act as auxiliary role in taking care of grandchildren. Affected by Chinese traditional concepts, generally women do housework and bring their children, while men work outside, women bear more care responsibility in their families [27]. So will this kind of gender difference of grandchild care will lead to the different effects of grandchild care on the depression of the older adults? Is female older adults more likely to be affected by grandchild care?

The rate of widowhood of older adults is relatively high. In life and emotionally, the older adults who don’t have a spouse, especially widowed, lack a spouse as the object of confession in life, often face a higher risk of depression than the older adults with a spouse [28, 29] . Grandchild care as a two-way emotional exchange between grandparents and grandchildren, reduces the loneliness of the grandparents, increases the opportunities for the grandparents to participate in society. For those older adults who do not have a spouse, grandchild care can make up for the lack of a spouse to a certain extent. This raises the question, compared to older adults with spouses, does grandchild care have varying impact on the depression of the older adults who don’t have a spouse? Therefore, this research further explores the effect of grandchild care on the depression of older adults of different genders and different marital status on the basis of previous studies.

### Grandchild care, intergenerational support from children and Chinese older adults depression

Taking care of grandchildren as a kind of social participation provides grandparents with stronger and more frequent contact with adult children, and make them have more opportunities to obtain the support of adult children [30]. Chinese grandparents also rely on caring their grandchildren in exchange for intergenerational support from their children in old age [31]. In other words, taking care of grandchildren increases the likelihood that the older adults will receive more intergenerational support from their adult children.

In previous studies, scholars have explored the influence of intergenerational support from children on the older adults depression . With the development of China’s economy, there are more ways for the older adults in China to obtain social support. However, influenced by Confucianism and traditional Chinese filial piety culture, intergenerational support from children is still the main source of life for the older adults in their later years [32]. And this family idea makes the intergenerational support from children have a very important protective effect on the mental health of the older adults [33].

According to the existing literature, scholars often only study the relationship between grandchild care and depression of the older adults, the relationship between intergenerational support from children and depression of the older adults, the relationship between grandchild care and intergenerational support from children [17] [34]. No scholar has ever discussed the combination of these three, and explored the relationship and action path among them. This article combines these three to propose a hypothesis: In the context of China's unique social and cultural environment and closely-connected intergenerational relationships, will grandchild care affect the depression of older adults by affecting the intergenerational support from their children?

Therefore, this research will explore the effect of grandchild care on the depression of Chinese older adults from two paths. One is whether the effect of grandchild care on the depression of Chinese older adults is different between different genders and marital status. The other is whether grandchild care affects the depression of the older adults through intergenerational support from children.

## Methods

### Sample selection

This research is based on the China Health and Retirement Longitudinal Study 2018(CHARLS2018) database to study the impact of grandchild care on the depression of Chinese older adults. The China Health and Retirement Longitudinal Study (CHARLS) is large-scale social survey hosted by National School of Development of Peking University. It has the characteristics of wide coverage, strong representativeness and rich survey content. The CHARLS National Baseline Survey was launched in 2011. The respondents of CHARLS2011 were 17,000 middle-aged and older adults aged 45 and above, from about 10,000 households, covering 150 county-level units and 450 village-level units. These samples would be tracked every two to three years [35]. The purpose of CHARLS sampling is to ensure the unbiased and representativeness of the sample. Sampling is carried out through four stages at the county(district)-village(residential)-household-individual level [36]. CHARLS used the PPS (Probability Proportional to Size) method to sample at two levels: county(district)-village(residential). First, 150 districts and counties were randomly selected, and 3 villages or communities were randomly selected from each of the above 150 districts and counties, and finally 450 villages/communities were obtained. In each village or community, CHARLS used a dedicated mapping software (CHARLS-GIS) designed and developed by itself to carry out on-site mapping and collect household information, randomly select 25-36 houses from the map, and determined the number of samples in each house. In addition, there was a filter section at the beginning of the CHARLS questionnaire, which could eliminate invalid samples. When studying the health and pension issues of middle-aged and older adults in China, it can provide rich and comprehensive information covering the respondent's family, health status and function, cognition and depression, medical care and insurance, pension, work and retirement. CHARLS database has good reliability and validity, and can well represent the actual situation of middle-aged and older adults in China. In recent years, it has been used by many scholars to conducted health-related research [35].

**Fig. 1:**
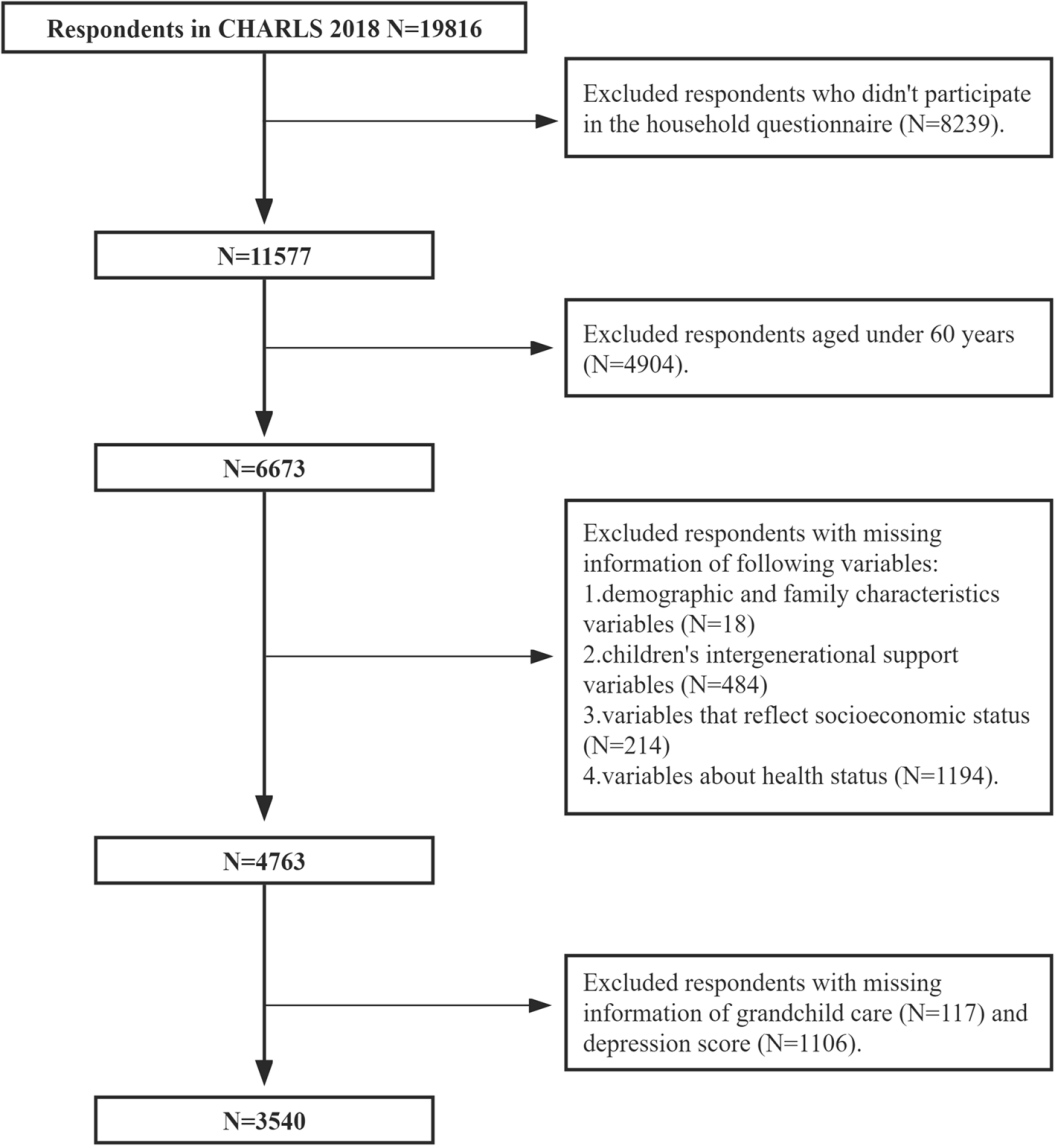
Respondents’ flow in the study

CHARLS2018 collected 19,816 respondents' information. Because the research content of this study involves household questionnaires and the research object is the older adults, 8239 respondents who did not participate in the household questionnaire are excluded, and 4904 respondents under the age of 60 are excluded, and further screening is carried out on this basis. Among the remaining respondents, 18 are missing information about demographics and family characteristics, 484 are missing information about intergenerational support from their children, 214 are missing information related to socioeconomic status, and 1194 are missing information about health status related information. In addition, 117 respondents who lack grandchild care information and 1106 lack depression scores are excluded (other questions can be answered by others if the respondent has limited ability, but others are not allowed to answer questions about depression of respondents, so there are many missing values ), 3540 respondents are finally included in our study, and the sample selection process is shown in Figure 1.The CHARLS database is approved by the Peking University Ethics Committee and is disclosed to the academic community, so no additional ethics approval is required.

### Variable description

#### Dependent variable

The dependent variable of this study is depression, and this variable is based on cognitive and depression part’s content of the CHARLS questionnaire. CHARLS uses the 10-item central depression scale (CES-D) modified by Andresen in 1994 to measure the depression of respondents. This scale has high reliability and validity, coupled with short answering time and high recovery rate. Therefore, it has great application potential in large-scale survey and research [37]. The scale consists of 10 items. By asking respondents how often the symptoms described by each item occurred in the past week, the four frequency options are”rarely or none”, “unusual”, “sometimes or half of the time”, and “most of the time”. In our research, the four levels of negative symptoms are assigned as 0, 1, 2, and 3 in turn, and two positive symptoms are assigned 3, 2, 1, and 0. The total score of the scale is between 0-30 points. The higher the score, the more serve the depression.

#### Independent variable

The independent variable is whether grandchild care is provided. According to CHARLS, the variable is measured by asking the respondent "Did you and your spouse spend time taking care of your grandchildren in the past year?", if the respondent’s answer is "Yes" , the older adults are considered to have provided grandchild care. If the answer is "No", they are deemed not to provide grandchild care, and are respectively assigned as 1 and 2 according to the questionnaire settings.

#### Control variables

The depression of the older adults is affected by many factors. The control variables included in this research include four categories, including variables of demographic and family characteristics, variables that reflect socioeconomic status, variables about the respondent’s health status , and intergenerational support from children variables.

Variables of demographic and family characteristics include gender, age, marital status, and number of children. Age is a continuous variable, calculated by subtracting the respondent’s birth year from 2018 (the year of the CHARLS2018 was conducted). Marital status is based on the six options in the questionnaire: “married and living with a spouse,” “married but temporarily not living with the spouse for work and other reasons,” “separated, no longer living together as a spouse,” “divorced,” “widowed,” and “never married,”are reclassified, the first three are "married" and assigned a value of 1, and the last three are "divorced\widowed\never married" and are assigned a value of 2.

A large number of studies have shown that socioeconomic status is the most powerful predictor of health, disease origin, and life span [38, 39]. The variables used in this study to reflect socioeconomic status include education, type of residence, type of medical insurance, and pension. Among them, education is classified as into four categories according to the population distribution and the questionnaire, including "elementary school and below", "junior school", "high school", and " College or above ". The type of residence is divided into urban and rural areas. The type of medical insurance corresponds to the questionnaire’s EA001, in which CHARLS divides the medical insurance into ten categories. For that the number of participants in the last six options is very small. Therefore, the types of medical insurance in this paper are redivided into five categories: Urban employee medical insurance, Urban and rural resident medical insurance, Urban resident medical insurance, New rural cooperative medical insurance and other insurance, and assign values of 1-5 in turn. Pension is measured by asking the respondent whether they are participating in pension insurance, and the answer "yes" is assigned a value of "1", and "no" is assigned a value of "2".

In recent decades, the rate of chronic diseases among the older adults has gradually increased, and the long-term and multi-disease coexistence of chronic diseases easily make patients have a higher risk of depression [40]. With the increase of age, the organs of the older adults decline and their physical activity is restricted, which seriously affects their mental health [41]. Therefore, the health status variables of the respondents included in this paper are the number of chronic diseases and activities of daily living (ADL). The number of chronic diseases in this study is measured by asking the respondent whether they have 14 chronic diseases in turn. If the answer is yes, it is recorded as 1, otherwise it is 0. The total number of chronic diseases is obtained by adding these 14 diseases. ADL is measured according to the physical life scale in the questionnaire, that is, whether the respondent has difficulty in dressing, bathing, eating, getting up, going to the toilet, and controlling excretion. Any difficulty in any item is recorded as 1, and all six items are all no difficulty is counted as 0.

Intergenerational support from children is measured from two aspects, namely, economic support representing economic assistance and emotional support measured by contact frequency. Among them, the economic support provided by the children to their parents is measured by asking the respondent and the respondent’s spouse about the total amount of money and goods given by the children in the past year. In order to avoid the influence of extreme values, the value of this variable is added “1”and then logarithm (that is, ln(x+1)) in the empirical analysis of this paper. Emotional support is measured by the contact frequency. Respondents who do not live with their children determined the frequency of contact between the respondent and their children according to the questionnaire"How often do you communicate with children you do not live with?" This study redivided ten options of the questionnaire into five options, which are "hardly", "seldom", "almost monthly", "almost weekly" and "almost every day". By the increasing contact frequency ,this variable is assigned a value of 0-4 in turn. Besides,we assign a value of 5 to respondents who live with their children. Therefore, the value range of this variable is 0-5, that is, the larger the value, the higher the contact frequency with their children.

### Research methods

This study first uses the ols model to perform regression analysis on the full samples to study the effect of grandchild care on the depression of the Chinese older adults, and then explores the gender difference in the the effect of grandchild care on the depression, and the marital status difference in the the effect of grandchild care on the depression. Finally, a mediating effect test is used to analyze the mediating effect of intergenerational support from children between grandchild care and depression of the older adults.

## Results

### Descriptive statistics

Table 1 is the basic characteristics of the respondents in this study, and the respondents are described separately according to gender and marital status. In terms of depression, the overall depression score of the older audlts in China is 10.15, which has reached mild depression according to the standard [42]. For the full sample, about 40% provide grandchild care. In addition, according to Table 1, it can also be seen that the respondents’ level of education is low, and the proportion of pensioners is small. The respondents’ residence is mainly rural and the type of medical insurance mainly they participate in is new rural cooperative medical insurance.

**Table 1:** Descriptive statistics

Variable	All(N=3540)	Gender		Marital status	
		Man (*N*=1677)	Woman (*N*=1863)	Married (*N* = 2492)	Divorced\widowed\never married (*N* = 1048)
mean(SD)					
Depression	10.15(6.97)	9.01(6.48)	11.18(7.23)	9.75(6.77)	11.11(7.33)
Age	69.41(6.60)	69.73(6.70)	69.13(6.50)	68.12(5.99)	72.49(6.97)
Numbers of Chronic diseases	0.88(1.13)	0.89(1.15)	0.88(1.11)	0.88(1.11)	0.89(1.18)
Ln(econimic support)	7.07(2.77)	7.05(2.85)	7.09(2.71)	7.13(2.78)	6.94(2.75)
Numbers of Children	3.46(1.65)	3.42(1.62)	3.49(1.67)	3.26(1.54)	3.92(1.80)
N(%)					
Care					
Provide	1383(39.07)	662(39.48)	721(38.7)	1076(43.18)	307(29.29)
Not provide	2157(60.93)	1015(60.52)	1142(61.3)	1416(56.82)	741(70.71)
Contact frequencey					
Seldom	32(0.90)	21(1.25)	11(0.59)	20(0.80)	12(1.15)
Hardly	576(16.27)	301(17.95)	275(14.76)	437(17.54)	139(13.26)
Almost every month	485(13.70)	249(14.85)	236(12.67)	367(14.73)	118(11.26)
Almost every week	583(16.47)	288(17.17)	295(15.83)	433(17.38)	150(14.31)
Almost every day	706(19.94)	310(18.49)	396(21.26)	508(20.39)	198(18.89)
Living together	1158(32.71)	508(30.29)	650(34.89)	727(29.17)	431(41.13)
Gender					
Male	1677(47.37)			1387(55.66)	290(27.67)
Female	1863(52.63)			1105(44.34)	758(72.33)
Marital status					
Married	2492(70.40)	1387(82.71)	1105(59.31)		
Divorced\widowed\never married	1048(29.6)	290(17.29)	758(40.69)		
Education					
Primary school or below	2678(75.65)	1110(66.19)	1568(84.17)	1807(72.51)	871(83.11)
Junior high school	535(15.11)	345(20.57)	190(10.20)	427(17.13)	108(10.31)
Senior high school	279(7.88)	192(11.45)	87(4.67)	225(9.03)	54(5.15)
College or above	48(1.36)	30(1.79)	18(0.97)	33(1.32)	15(1.43)
Residence					
Urban areas	929(26.24)	406(24.21)	523(28.07)	635(25.48)	294(28.05)
Rural areas	2611(73.76)	1271(75.79)	1340(71.93)	1857(74.52)	754(71.95)
Pension					
Participate in	727(20.54)	414(24.69)	313(16.8)	544(21.83)	183(17.46)
Not participate in	2813(79.46)	1263(75.31)	1550(83.2)	1948(78.17)	865(82.54)
Types of medical insurance					
Urban employee medical insurance	513(14.49)	284(16.94)	229(12.29)	377(15.13)	136(12.98)
Urban and rural resident insurance	446(12.60)	202(12.05)	244(13.10)	309(12.40)	137(13.07)
Urban resident medical insurance	161(4.55)	58(3.46)	103(5.53)	107(4.29)	54(5.15)
New rural cooperative medicial insurance	2329(65.79)	1075(64.10)	1254(67.31)	1631(65.45)	698(66.6)
Other	91(2.57)	58(3.46)	33(1.77)	68(2.73)	23(2.19)
ADL					
No difficulty	2464(69.60)	1231(73.40)	1233(66.18)	1805(72.43)	659(62.88)
Difficulty	1076(30.40)	446(26.60)	630(33.82)	687(27.57)	389(37.12)

According to Table 1, it can be seen that female score of depression is 2.17 higher than male’s on average, and the difference of the depression of different genders older adults is statistically significant (P<0.001). Compared with male, female’s connection with the children is closer. After classification by marital status, the average depression score of the older adults who don’t have a spouse is 1.36 higher than that of the older adults with a spouse, and this difference is also significant at the level of P<0.001. Compared with the married older adults, the older adults who don’t have a spouse have the characteristics of providing less grandchild care, higher probability of living together with children.

### Regression analysis

#### Full sample regression analysis

Table 2 is a full-sample regression of the effect of grandchild care on the depression of the older adults. Model 1 includes variables of demographic and family characteristics, variables that reflect socioeconomic status, and variables about the health status of the respondents. Model 1 shows that whether to provide grandchild care has a significant impact on the depression of the older adults. Model 2 adds intergenerational support variables . It can be found that both emotional support and economic support have a significant impact on the depression of the older adults. After including intergenerational support from children variables, the significance of grandchild care is weakened. This result suggests the existence of the mediating effect of intergenerational support.

**Table 2:** Regression analysis

Depression	Model1	Model2
Care	0.585**	0.452*
	(0.235)	(0.235)
Gender	1.386***	1.447***
	(0.231)	(0.230)
Age	-0.106***	-0.100***
	(0.0205)	(0.0204)
Marital status	0.715***	0.696***
	(0.259)	(0.259)
Numbers of Children	0.202***	0.266***
	(0.0764)	(0.0771)
Education	-0.687***	-0.662***
	(0.179)	(0.179)
Residence	0.720**	0.599*
	(0.310)	(0.310)
Pension	1.142***	1.209***
	(0.389)	(0.388)
Types of medical insurance	0.218*	0.231*
	(0.119)	(0.119)
Number of Chronic Disease	0.834***	0.818***
	(0.0964)	(0.0962)
ADL	3.829***	3.827***
	(0.239)	(0.238)
Contact frequencey		-0.245***
		(0.0735)
Ln(Ecosupport)		-0.169***
		(0.0392)
Constant	7.826***	9.412***
	(1.613)	(1.636)
Observations	3,540	3,540
R-squared	0.161	0.168

Standard errors in parentheses

*** *p*<0.01, ** *p*<0.05, * *p*<0.1

**Fig. 2:**
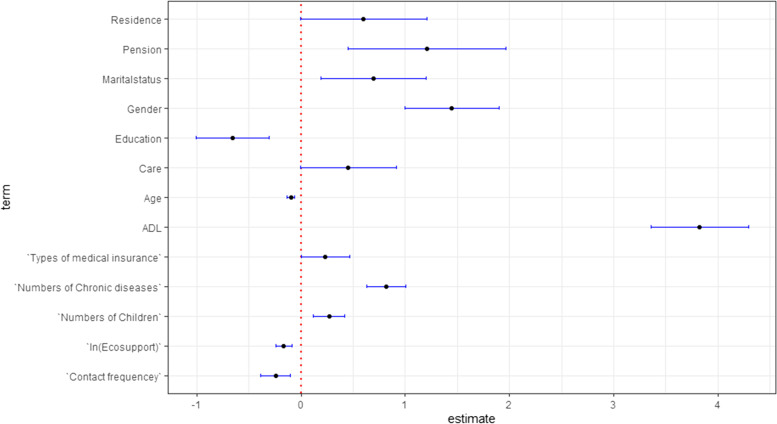
Visualization of regression coefficients

In order to show the relationship between each variable and the dependent variables more clearly, we draws Figure 2 using R. Figure 2 shows the visualization of the regression coefficients of Model 2 in Table 2. Through this figure, we can see that the confidence intervals of all coefficients do not contain 0. Grandchild care and other control variables have a significant impact on the depression of the respondents. The more intergenerational support children provide, the higher the socioeconomic status, the better their health, the lower the depression scores of the older adults, which is consistent with the results of previous studies [43, 44]. Among them, ADL has the greatest impact on the depression score of the older adults.

#### Regression analysis of different genders

Table 3 shows the regression results by different genders. The regression results of the effect of grandchild care on the depression of male older adults show that not only model 1 but also model 2 which the intergenerational support from children variables are added, grandchild care does not have a significant impact on the depression of Chinese male older adults. For Chinese female older adults, according to the results of Model 3, grandchild care has a significant impact on the depression. After the intergenerational support from children variables are added to Model 4, the coefficient of grandchild care becomes smaller.

**Table 3:** Regression analysis by different genders

Depression	Male		Famale	
	Model1	Model2	Model3	Model4
Care	0.0305	-0.0827	1.095***	0.919***
	(0.323)	(0.324)	(0.339)	(0.340)
Age	-0.0814***	-0.0775***	-0.129***	-0.121***
	(0.0273)	(0.0272)	(0.0306)	(0.0305)
Marital status	0.694*	0.553	0.808**	0.891***
	(0.408)	(0.409)	(0.342)	(0.341)
Numbers of Children	0.201*	0.265**	0.190*	0.246**
	(0.105)	(0.107)	(0.111)	(0.111)
Education	-0.585***	-0.540**	-0.888***	-0.905***
	(0.212)	(0.212)	(0.316)	(0.315)
Residence	0.308	0.201	0.979**	0.863*
	(0.429)	(0.431)	(0.449)	(0.448)
Pension	1.612***	1.629***	0.580	0.691
	(0.489)	(0.488)	(0.626)	(0.624)
Types of medical insurance	-0.0127	0.0188	0.481***	0.471***
	(0.155)	(0.155)	(0.183)	(0.182)
Number of Chronic Disease	0.789***	0.772***	0.876***	0.868***
	(0.131)	(0.131)	(0.141)	(0.140)
ADL	3.548***	3.563***	4.008***	3.986***
	(0.340)	(0.339)	(0.336)	(0.335)
Contact frequencey		-0.109		-0.377***
		(0.0995)		(0.108)
Ln(econimic support)		-0.183***		-0.164***
		(0.0533)		(0.0575)
Constant	9.037***	10.54***	11.17***	13.10***
	(2.080)	(2.129)	(2.263)	(2.296)
Observations	1,677	1,677	1,863	1,863
R-squared	0.128	0.134	0.156	0.165

Standard errors in parentheses

*** *p*<0.01, ** *p*<0.05, * *p*<0.1

#### Regression analysis of different marital status

Table 4 shows the regression results of different marital status. For the older adults who are married, not only model one but also model two with intergenerational support from children variables, whether to provide grandchild care can not have a significant impact on their depression. For older adults who don’t have a spouse, whether to provide grandchild care significantly affects their depression. After the intergenerational support from children variables are added in Model 4, grandchild care still has a significant impact on the depression, but the regression coefficient of grandchild care becomes smaller and the significance is reduced.

**Table 4:** Regression analysis by different marital status

Depression	Married		Divorced\widowed\never married	
	Model1	Model2	Model3	Model4
Care	0.337	0.195	1.273***	1.175**
	(0.268)	(0.268)	(0.483)	(0.486)
Age	-0.100***	-0.0947***	-0.116***	-0.111***
	(0.0250)	(0.0249)	(0.0362)	(0.0363)
Gender	1.377***	1.402***	1.594***	1.753***
	(0.262)	(0.261)	(0.481)	(0.485)
Numbers of Children	0.293***	0.376***	0.0470	0.0759
	(0.0935)	(0.0947)	(0.134)	(0.135)
Education	-0.646***	-0.630***	-0.851**	-0.801**
	(0.201)	(0.200)	(0.388)	(0.388)
Residence	0.587	0.407	1.092*	1.076*
	(0.364)	(0.365)	(0.591)	(0.590)
Pension	1.621***	1.674***	-0.398	-0.313
	(0.441)	(0.439)	(0.811)	(0.810)
Types of medical insurance	0.0216	0.0465	0.757***	0.766***
	(0.137)	(0.136)	(0.240)	(0.241)
Number of Chronic Disease	0.758***	0.728***	0.993***	1.010***
	(0.114)	(0.114)	(0.181)	(0.181)
ADL	3.779***	3.786***	3.879***	3.859***
	(0.285)	(0.284)	(0.442)	(0.441)
Contact frequencey		-0.294***		-0.114
		(0.0858)		(0.143)
Ln(econimic support)		-0.177***		-0.177**
		(0.0457)		(0.0765)
Constant	8.328***	10.21***	9.498***	10.29***
	(1.987)	(2.015)	(3.192)	(3.218)
Observations	2,492	2,492	1,048	1,048
R-squared	0.152	0.160	0.172	0.177

Standard errors in parentheses

*** *p*<0.01, ** *p*<0.05, * **p**<0.1

### Mediation effect test

This research further uses the full sample to explore the mediating effects of intergenerational support from children. Model 1 examines the mediating effect of children’s emotional support, and Model 2 examines the mediating effect of children’s economic support. Both models control the variables of demographic and family characteristics, variables that reflect socioeconomic status and variables about the health status of the respondents. In this research, the bootstrap method is used to repeat sampling 1000 times, and the results are shown in Table 5. It can be seen from Table 5 that the confidence interval of the children's emotional support’s indirect effect does not include 0 ,so it is considered that there is a mediating effect [45], and the size of the mediating effect is 0.074. The confidence interval of the children's economic support’s indirect effect does not contain 0, and there is also an mediating effect with a magnitude of 0.047.

**Table 5:** Analysis of mediation effect

Mediator effect model	β (95% CI)	SE	Z	P
Model1				
Indirect effect	0.074** ( 0.019, 0.129)	0.028	2.62	0.009
Direct effect	0.511**(0.057,0.965)	0.224	2.28	0.023
Model2				
Indirect effect	0.047** (0.010,0.085)	0.019	2.49	0.013
Direct effect	0.537**(0.064,1.010)	0.241	2.23	0.026

Control variables in both model1 and model2: Age, Gender, Numbers of Children, Education, Residence, Pension, Types of medical insurance, Number of Chronic Disease, ADL, Numbers of chronic diseases

*** *p*<0.01, ** *p*<0.05, * *p*<0.1

**Fig. 3:**
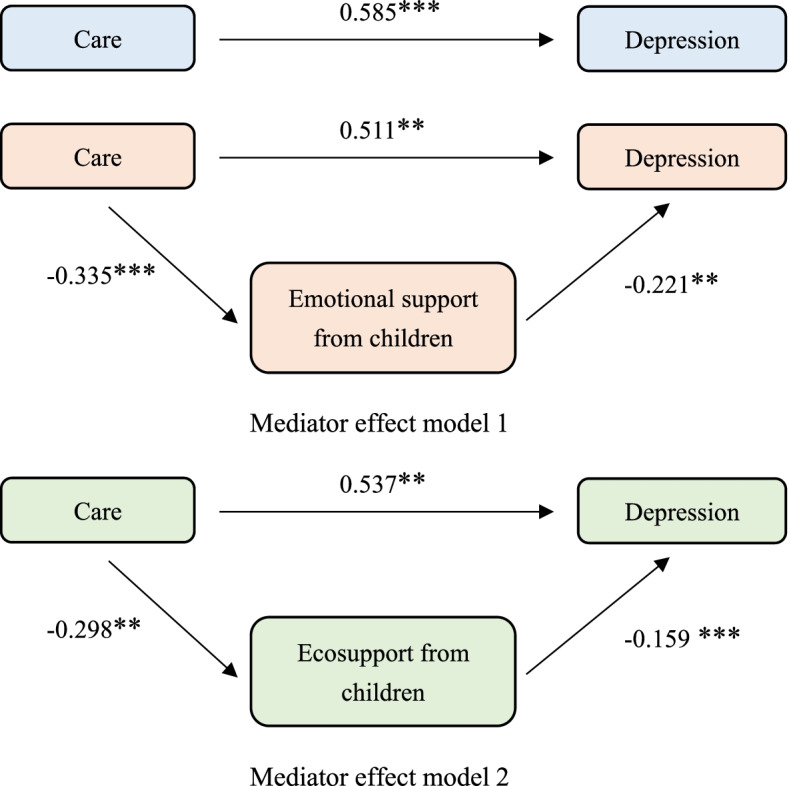
Intermediary effect roadmap

In order to show the influence path of intergenerational support more clearly, we have drawn Figure 3.It can be seen from the figure 3 that when other variables are controlled in the model and no intermediary variables are included, grandchild care has a significant impact on the depression of the older adults (p<0.001). When the contact frequency is included as an intermediary variable into the model, whether to provide grandchild care still has a significant effect on depression of the older adults (p<0.05). Meanwhile the effect of grandchild care on children’s emotional support is significant (p<0.001), and the effect of children’s emotional support on depression is significant too (p<0.05).

When children’s economic support is included as an intermediary variable into the model, whether to provide grandchild care has a significant effect on the depression of the older adults (p<0.05). In this path, the effect of grandchild care on children's economic support is significant (p<0.05) and children's economic support’s effect on depression is significant too (p<0.001).

Therefore, the mediation variables of both paths have a partial mediation effect .It can be considered that the older adults who provide grandchild care can get more emotional support and economic support from their children, thereby making the older adults less depressive. The intergenerational support from children plays a mediating role between grandchild care and depression of Chinese older adults.

## Discussion

Based on the CHARLS2018 database, this paper studies the effect of grandchild care on the depression of Chinese older adults, and uses the Bootstrap method to analyze the mediating effect of intergenerational support from children. The regression results of the full sample show that providing grandchild care has a significant postive effect on the depression of Chinese older adults. After adding intergenerational support variables, whether to provide grandchild care has a weaker effect on the depression. The regression results of different genders show that grandchild care has a significant impact on the depression of female older adults. The regression results of different marital status show that grandchild care has a significant impact on the depression of the older adults who don’t have a spouse.

The mediating effect of intergenerational support from children is tested on the full sample. The results show that intergenerational support from children has a significant mediating effect between grandchild care and the depression of the older adults. Providing grandchild care affects the depression of Chinese older adults through increasing their intergenerational support from children.

According to the research in this paper, depression is one of the health threats of Chinese older adults. The average depression score of Chinese older adults has reached the level of mild depression, and taking care of grandchildren can help older adults resist some of the risks of depression. Jing Zhang also used the CHARLS database to conduct research and explored the relationship between caring for grandchildren and the loneliness of the older adults, and found that caring for grandchildren can reduce the possibility of Chinese older adults feeling lonely [46]. Fengyan Tang's research found that compared with those who did not take care of their grandchildren, the older adults who provided grandchild care in China had fewer depressive symptoms, but the intensity of care should be moderate [47]. Seung-won Emily Choi considered the characteristics of family structure when studying grandchild care and depression of the older adults in China, and found that taking care of grandchildren in intergenerational families has a lower degree of depressive symptoms than the older adults who do not take care of their grandchildren [48]. The results of this study and previous studies on grandchild care and health status of Chinese older adults tend to the role expansion theory, that is, grandparents have obtained significant positive benefits by participating in grandchild care. In the context of China’s aging society, grandchild care effectively relieves the pressure on adult children, taps the human resources of the older adults. The caregiver of grandchildren is an important social role of the older adults and enhances the sense of mission of the older adults [31]. Taking care of grandchildren can increase the chances of social interaction, contacting with relatives and friends, and communication with children, reduce the loneliness of grandparents. At the same time, raising grandchildren is also a very fulfilling thing, which brings self-confidence to grandparents. These are all conducive to reducing their risk of depressive symptoms.

Di Gessa, Giorgio and Bordone, Valeria used the SHARE to study the relationship between European grandchild care and mental health. The results also point to the role expansion theory, that is, taking care of grandchildren have a positive effect on mental health. In the United States, according to the results of Jan Blustein's research, grandparents whose grandchildren are at home are more likely to have symptoms of depression than those whose grandchildren are not at home [49]. Minkler, M found that the older adults who provided primary care for their grandchildren are twice as likely to suffer from depression as those who do not [50]. Whether the relationship between caring for grandchildren and depression points to role strain theory or role expansion theory, perhaps there is a great correlation between cultural background.

Our research finds that the effect of grandchild care on the depression of Chinese older adults of different genders is different, and it has a significant effect on the depression of female older adults, but it does not have a significant effect on the depression of male older adults. Previous studies on the social participation of the older adults in China have shown that male older adults in China are more involved in public sphere activities than females, and less involved in family care and housework activities. Female older adults usually focus on family life [51, 52] . Based on traditional Chinese concepts, scholars have explored the relationship between family responsibilities and depression from the perspective of gender differences. The results show that females are more likely to bear family responsibilities, and they also appear to be more likely to suffer from depression [53]. Grandchild care is a kind of family responsibility, although many male older adults participate, they take less responsibility, and often play a supporting role. Less taking care of responsibilities leads to the result that taking care of grandchildren does not have a significant effect on depression. Therefore, whether to provide grandchild care is not one of the influencing factors of the depression of Chinese male older adults.

The effect of grandchild care on the depression of Chinese older adults is also different among the older adults with different marital status. Marriage is the most important interpersonal relationship for adults in China, and widowed, divorced or never married means the absence of this important social network [54]. Marital status is an important factor affecting depression in the older aduts. Without the company of a spouse, they face a greater risk of depression [55]. Compared with the older adults with a spouse, not having a spouse means insufficient emotional comfort and lack of access to care in old age. Taking care of grandchildren brings about emotional interactions between grandparents and grandchildren, makes the older adults broaden social networks and have more opportunities for social participation. More social activities, for those who don’t have a spouse, to a certain extent make up for the lack of emotional comfort and social networks, which has a positive impact on the mental health of widowed, divorced and never married older adults. However, grandchild care does not have a significant impact on the depression of married Chinese older adults. Previous research have proved that the protection mechanism of marriage plays a positive role in coping with depression during old age [17, 29]. The presence of a spouse can resist some potential risks of depression of the older adults and it can also weaken the effect of some factors that improve depression. Providing grandchild care as a potential factor in improving depression does not have a significant improvement effect on older adults with spouses. Therefore, whether to provide grandchild care can not have a significant impact on the depression of married older adults.

Qinghong He pointed out that parents provided grandchild care, and children also provided more support to their parents according to intergenerational exchange theory [30]. However, the role of intergenerational support from children due to grandchild care had not been further explored in previous studies. Our research finds that intergenerational support from children plays an intermediary role between grandchild care and the depression of the older adults. Grandchild care for the older adults is not only social participation in the ordinary sense, but also assistance to children, which can reduce the burden of care for adult children and allow them to work with peace of mind. Children visit their parents with economic support because they are grateful for their help in taking care of grandchildren. Grandparents may think it is better than no visit and no economic support from their children. This kind of emotional and economic support helps to make up or even strengthen the intergenerational bond, thereby enhancing the intergenerational relationship between children and the older adults, allowing the older adults to have a sense of happiness, and achieve the improvement of depression of the older adults. It is a win-win for children’s families and grandparents’ families.

In recent years, China has gradually liberalized childbirth, and social support is seriously insufficient in childcare. Raising children has caused considerable pressure on the family and society. The grandchild care in China should be paid attention to and valued. At present, more and more women in China are participating in social labor. For many women of the right childbearing age, the contradiction between family and work is prominent, and the willingness to bear children is low [31]. On the basis of the older adults have the ability to take care and full respect for their willingness to provide grandchild care, society and families should encourage the older adults to participate in taking care of their grandchildren, encourage the older adults to raise their grandchildren scientifically, and provide training related to infant and childcare. This not only eases the burden of care for the children's family, increases women's willingness to bear children, but also makes the grandparents enjoy the family happiness of children and grandchildren. At the same time, for the older adults caring for their grandchildren, adult children should give them more intergenerational support. By increasing intergenerational connections and economic support, depression of the older adults can be improved to a greater extent, and the children can play the role of filial children better.

The innovation of this paper is to use a large national database to verify the effect of grandchild care on the depression of the older adults in China, and then classify the older adults by gender and marital status, and explore the effect of grandchild care on the depression of different older adults groups. On this basis, we further explored the mediating role of intergenerational support from children between grandchild care and the depression of the older adults, and finds that grandchild care can improve depression of the older adults by increasing intergenerational support from their children.

At the same time, this research also has certain limitations. First of all, this research uses cross-sectional data and cannot explore the causal relationship between grandchild care and the depression of Chinese older adults. Secondly, when exploring the impact of grandchild care on the depression of the older adults, the measurement and definition of the intensity of grandchild care has not yet been unified. Therefore, we only consider whether grandchild care is provided, but not consider the intensity of grandchild care. Finally, the characteristics of grandchildren are also important factors that affect the depression of the older adults. However, our research haven’t include the characteristics of grandchildren as control variables in the model due to the inability to obtain information about the grandchildren.

## Conclusion

By analyzing the results of the research, we conclude that grandchild care has an impact on the depression of the older adults in China, and the intergenerational support from children plays a mediating role between grandchild care and depression. Society should encourage capable older adults to participate in caring for grandchildren, especially female older adults and older adults who don’t have a spouse. Children should also provide more intergenerational support to the older adults who help them take care of their children. Grandchild care and intergenerational support from children both play an important role in promoting healthy aging and alleviating depression of the older adults.

## Data Availability

The database (CHARLS2018), which is used in this paper, is publicly available. (http://charls.pku.edu.cn/en).

## Abbreviations

ADL: Activities of daily living
CHARLS: China Health and Retirement Longitudinal Study
